# Barriers and enablers of post-COVID-19 acute care follow-up in Nigeria from service providers’ perspective: a nominal group technique

**DOI:** 10.1186/s12913-024-11032-w

**Published:** 2024-04-29

**Authors:** Justus Uchenna Onu, Iorhen Akase, Justice Ohaka, Ibrahim Musa Kida

**Affiliations:** 1 Department of Mental Health, Nnamdi Azikwe University, Nnewi, Anambra State Nigeria; 2 Department of Internal Medicine, College of Medicine, University of Lagos, Lagos, Nigeria; 3 Department of Community Medicine, River State University, Port-Harcourt, River State Nigeria; 4 Department of Internal Medicine, University of Maiduguri, Maiduguri, Borno State Nigeria

**Keywords:** Barriers, Enablers, Post-COVID condition, Follow-up, Nigeria

## Abstract

**Background:**

Despite modest efforts to study and document the complications that arise after acute treatment of patients with coronavirus disease, its ramifications and regional variations are yet to be clearly understood. Progress in sub-Saharan Africa, notably Nigeria, has been impeded by patient disengagement from care and insufficient or non-existent follow-up arrangements. The aim of this study was to describe the barriers and enablers for follow-up services after discharge from COVID-19 care pathway in Nigeria.

**Methods:**

Seventeen experts involved directly in the care of patients with COVID-19 participated in brainstorming using the nominal group technique during a national workshop to review the new guidelines for COVID-19 case management in Nigeria. Participants discussed the barriers and facilitators of post-acute care follow-up of patients discharged from COVID-19 pathway and ranked their recommendations to arrive at three major factors per question.

**Results:**

Participants were mostly middle aged and predominantly clinicians. The top three barriers were patients’ perception of their symptom severity, lack of organizational clarity/structure/policies on follow-up care after discharge, and financial constraints. Similarly, participants identified providers’ initiated education on the reasons for follow-up at discharge, written organizational policies/structure and clarity and free follow-up services as the top three facilitators.

**Conclusion:**

This study has enumerated barriers to follow-up care after discharge patients with coronavirus disease and highlighted providers, institutional and governmental responses that will facilitator follow-up care after discharge of patients with COVID-19. The implication is that, there is need for clear institutional guidelines for tracking and documenting post-COVID condition. In the future, it would be necessary to assess the achievements and shortcomings of post-COVID condition tracking in Nigeria through the use of implementation science outcomes.

## Background

The Covid-19 pandemic has necessitated rapid responses from healthcare systems across the globe [1]. As of the second quarter of 2023, the plague had ravaged humanity with over 768 million confirmed cases and over seven million fatalities worldwide [1]. Although a lot of information regarding acute symptoms and therapeutic care has been gathered and examined, little is known about the circumstances following discharge.

In response to this, the World Health Organization (WHO) created a clinical case definition for post-COVID-19 condition via Delphi approach that contains 12 dimensions, and is usable in all settings, to better understand the emerging disorders after acute treatment [2]. Post COVID-19 condition is said to occur when individuals with a history of probable or confirmed SARS CoV-2 infection, usually three months from the onset of COVID-19, present with symptoms that last for at least two months and cannot be explained by an alternative diagnosis [3].

Majority of persons who contract COVID-19 fully recover; however, current evidence suggests that approximately 10–20% persons go on to experience a range of mid and long-term effects after recovery from the initial illness [2]. A recent meta-analysis of studies with comparatively longer observation periods (12 months and beyond) found that the prevalence was less than 1% in non-hospitalized patients, 11% in hospitalized patients and doubled in patients admitted to the intensive care unit [4]. The most prevalent symptoms were fatigue and dyspnea with a pooled prevalence ranging from 27 to 58% [5]. Sleep disturbance, cough, anosmia/ageusia, fever, myalgia, chest pain, and headache were among the other post-COVID-19 symptoms [5]. Apart from the physical manifestations, anxiety and depression were also common, with rates ranging from 8 to 53% [5]. The identified significant risk factors were female gender with any symptom, with mental symptoms, with fatigue and acute disease severity with pulmonary symptoms [6]. In Africa, one study reported a prevalence of post-COVID-19 condition of 82.1% at one month and 66.7% at six months [7].

However, prior studies have indicated that follow-up for chronic illnesses can be challenging [8, 9]. Disengagement from care is sometimes frequent due to a number of factors that impact healthcare services in sub-Saharan Africa (SSA), including insufficient healthcare infrastructure and out-of-pocket expenses [10, 11]. With no specified protocol in many institutions about follow-up care of COVID-19 patients after acute symptoms, it is not clear if there are comprehensive post-care packages for patients after discharge in Nigeria. An enquiry into the barriers and enablers to follow-up from the perspective of case managers has become imperative. Case managers were selected on the basis of their direct experience in providing day-to-day care, including follow-up care, for patients with the condition. They also played crucial role in the State and National COVID-19 response teams.

Early in the pandemic, many authors reported on the facilitators and barriers of different services, such as the uptake of COVID-19 vaccination [12], routine care during the pandemic [13], and adherence to COVID-19 preventive measures [14]. These studies were centered on the events during the peak of the pandemic. To the best of our knowledge, there have been no studies particularly examining the barriers and enablers of follow-up services for post-COVID-19 conditions in SSA. The purpose of this study was to explore the barriers and enablers of follow-up care after discharge from COVID-19 acute care pathway in Nigeria.

## Methods

### Study design and setting

The study utilized Nominal Group Technique (NGT) which is both qualitative and quantitative. The NGT technique also known as the consensus method, is a structured approach to group brainstorming that promotes participation from all members of the group [15]. It works effectively for problem identification, coming up with solutions, and making decisions [15]. We utilized the opportunity of the National Training of Trainers (NToT) meeting organized by the Clinton Health Access Initiative (CHAI) at Abuja, Nigeria and supported by the Federal Ministry of Health to carry out this study.

### Sample size and study population

Seventeen healthcare workers and administrative staff mostly from the COVID-19 state teams (i.e., public health emergency, case management, mental and psychosocial support, infection prevention and control, communication, laboratory, logistics and surveillance units) from selected states in Nigeria participated in the study. Majority of the participants were selected from the case management pillar. A total population sample was utilized. All the participants agreed to take part in the process.

### Procedure

At the preliminary phase, the investigators prepared the questions for the NGT to answer the research questions. Logistics such as the venue, flipcharts and sitting arrangement were provided. The stages for the NGT is described below:

#### Introduction and explanation (stage 1)

The facilitator welcomed the participants and explained in details the purpose and process of the meeting. Thereafter, the participants were split into three groups of 5–6 members with a group leader who served as a facilitator for the group.

#### Silent generation of ideas (stage 2)

Everyone in the group was requested to make a list of the things that help or hinder the provision of follow-up services after discharge from acute COVID-19 treatment and record them on the allocated paper. At this stage, the group facilitator advised each participant to keep their ideas to themselves and not to consult anybody else. This stage lasted for about 10 minutes.

#### Round-Robin recording of ideas (Stage 3)

Each group leader invited the participants to share the ideas generated and each idea was recorded on a flipchart. This process continued until all ideas were recorded and all participant reached. The ideas were written down as stated by the participants, alterations in the wordings was only down with the permission of the participant that volunteered the idea. The facilitator for each group encouraged the members to write down new ideas that arose as others share. No debate on the ideas generated during this phase which lasted for 20 minutes.

#### Group discussion (stage 4)

Participants were invited to seek verbal explanation or further details about any of the ideas that colleagues have produced that may not be clear. The facilitator ensured that each person was allowed to contribute and that discussion of all ideas was thorough without spending too long on a single idea. The members of each group were allowed to suggest new items for discussion and combine items into categories, but no ideas was eliminated. This stage took about 45 minutes.

#### Ranking and voting (Stage 5)

Each group utilized both the ranking and rating systems to select top five ideas. In this system, each idea raised were ranked from 1 to 5 and assigned a score ranging from 5 to 1. For instance, an idea received a score of 5 if a participant ranked it as 1, and so on. The top five facilitators and barriers were ranked independently by each group member. The scores generated enabled each group to come up with their top five ideas which was recorded for presentation in the plenary. This took about 10 min.

A final master list of barriers and facilitators was created during the plenary session, after the top five ideas from each subgroup were noted on the flipchart and duplicate ideas were combined. A final vote to determine the top five barriers and facilitators was held after a lengthy discussion among the wider group.

### Data analysis

The socio-demographic and professional characteristics of the participants were described using summary statistics such as median and interquartile range for age. Frequency counts and percentages were used for categorical variables. Details of the ranking, rating and voting process is as described in the stage 5 above.

## Results

Table 1 shows the demographic and professional characteristics of the study participants. Out of the 17 participants, there were 12 males and five females. The participants were predominantly of middle age with median age of 43 years. The majority of the participants were medical doctors (76.5%) who treated patients with COVID-19.

**Table 1:** Demographic and professional characteristics of the participants

Variables	Frequency (%)	Median (IQR)
**Age (years)**		42.50 (14.75)
**Gender**		
Male	12 (70.6)	
Female	5 (29.4)	
**Professional status**		
Medical doctors	13 (76.5)	
Laboratory scientist	1 (5.9)	
Administrative staff	3 (17.6)	

### Question 1: what are the (top 5) challenges you think you face in following up patients discharged from COVID-19 care pathway?

**Fig. 1:**
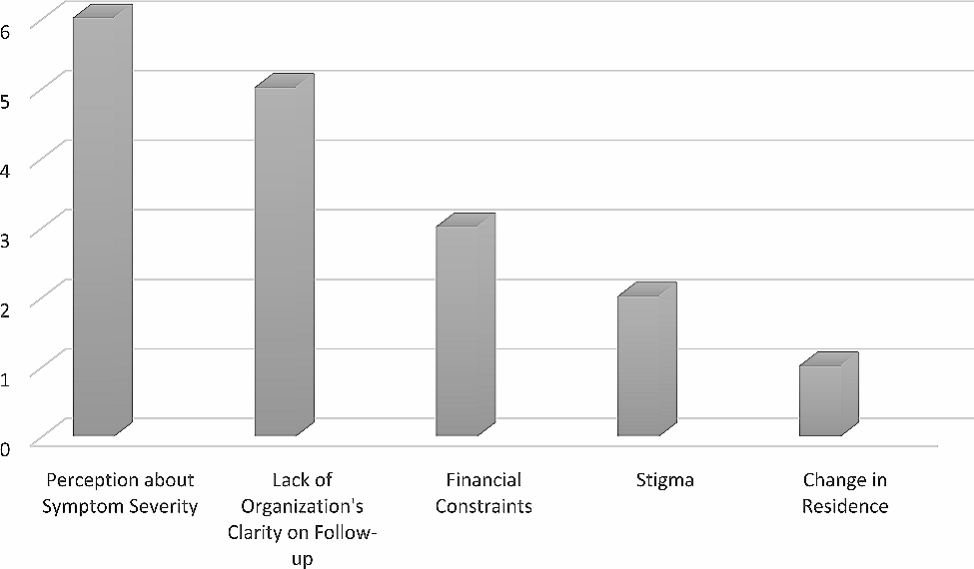
Ranking of the barriers to follow-up after acute care of COVID-19 patients

The participants’ ranked and rated the following top five barriers, namely; patients’ perception of the usefulness of follow-up and the seriousness of the post-COVID-19 symptoms, lack of institutional clarity about where and whom should continue with the follow-up care after discharge, financial constraints, stigma and change in the area of residence. This is shown in Fig. 1.

### Question 2: what are the potential solutions to the identified barriers to follow-up after discharge from COVID-19 care pathway?

**Fig. 2:**
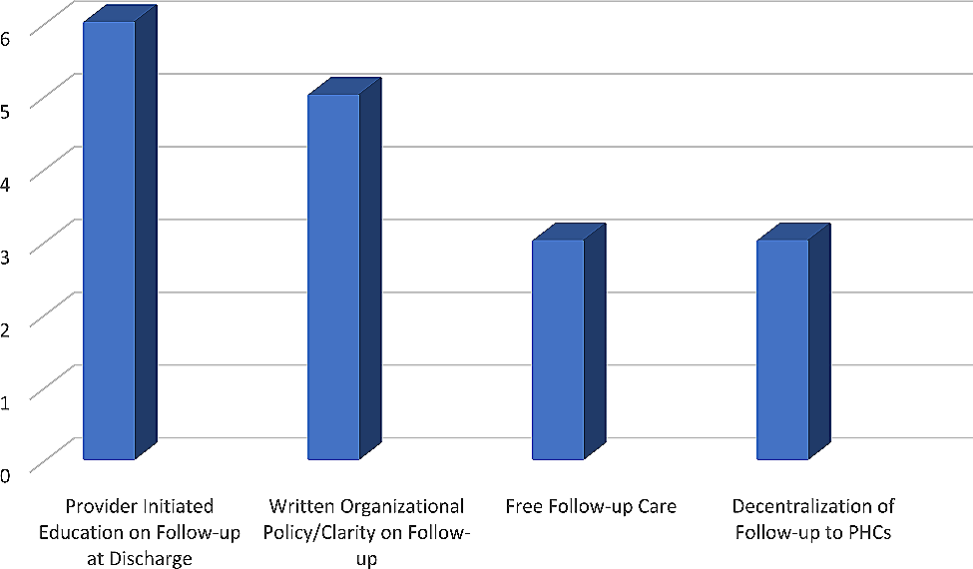
Participants’ ranking of the top facilitators to follow-up care after discharge of COVID-19 patients

In response to the above question, the participants identified and rated the following facilitators/solutions in the order of importance as shown in Fig. 2. They include: service providers’ initiated communication on the importance of follow-up after discharge, written institutional policy on follow-up, free follow-up services and decentralization of follow-up care to the primary health care settings.

## Discussion

The barriers and facilitators of follow-up of patients with COVID-19 after discharge from acute was highlighted in this qualitative study using nominal group technique.

To the best of our knowledge, it is the first paper in Africa to address the barriers and enablers of follow-up after COVID-19 acute care from the perspective of service providers using this design. The main takeaways from the findings are as follows: (1) the top three barriers identified were client perception of the severity of symptoms, lack of organization policy/structure/clarity on follow-up, and financial constraints; (2) the top three facilitators were provider-initiated education on follow-up services after discharge, clarity of organizational policy on follow-up, and offering provision of free follow-up service.

The top three barriers were identified as need factors (e.g., perception of symptom severity), organizational factors (e.g., lack of clarity of follow-up policy), and resource-related factors (e.g., financial constraints). This finding resonates with previous reports in the literatures [16–18]. For instance, Castro-Avila et al. [18]., in a recent study found that the funding complexities, lack of competence, and the communication gaps between the intensive care unit and community services were the common themes linked to barriers in providing follow-up services after discharge from COVID-19 acute care. Furthermore, unclear follow-up policies was a challenge, since more than 60% of general practitioners studied were not aware of the follow-up services offered by their respective institutions [18]. This is consistent with our findings, and the reasons for their similarity could stem from a shared methodology of using service providers to obtain information on barriers.

Similarly, in another study of barriers and facilitators of retention in chronic disease in Western Kenya, Rachlis et al. [16] reported that the major obstacles to continuity of care were personal drive, poor patient-provider relationship and lack of social support. Similarly, in Parkistan Abbas et al. [17]., found that patient’s doubt of diagnosis, inadequate help with physical symptoms, failure to provide essential information by providers, and unempathetic response to queries were the major barriers to follow-up.

In both studies [16, 17], retention in care was found to be facilitated by patient characteristics (such as level of motivation), the availability of enabling resources (such as financial support), and an accommodating healthcare environment. These facilitators identified in previous studies differ slightly from the provider-enabled patients’ education, clarity of organizational policies and free follow-up services enumerated in the index study. However, one recent report emphasized the usefulness of optimal service providers’ initiated communication in discharge readiness of patients after acute care of COVID-19 [19]. Methodological issues could be responsible for the modest variations. While the index study focused on the providers’ perspective alone for a relatively new disease (COVID-19), the two studies highlighted above utilized patients’, caregivers’ and healthcare providers’ perspective. One other facilitator in the index study was the decentralization of follow-up to Primary Healthcare centers (PHC). This is consistent with the robust reports in the literature that many diseases can be effectively managed in PHCs [20, 21].

Furthermore, the Nigerian health care systems must be taken into consideration while interpreting these results. The majority of the barriers and facilitators mentioned in the index study mirror current problems in the Nigerian healthcare system. For example, the participants highlighted the importance of financial constraints in limiting follow-up after discharge from the acute COVID-19 care pathway. They opined that free services will enhance attendance. This is consistent with the findings of Elugbadebo et al. [22]., who reported that the commonest reasons for discontinuation of care among out-patients were financial constraints and long distance to the hospital. This re-echoes the need to expand the coverage of the national health insurance policy. Currently, over eight out every 10 Nigerian pay for health services out-of-pocket due to poor coverage [11].

### Limitation

The use of only service providers may have limited the diversity of the viewpoints. An integrative synthesis of both patients’ and providers’ perspective may improve the diversity and convergence of opinions.

## Conclusion

The findings of this study show that organizational clarity, decentralization of care, structured education of the patient before discharge are needed to ensure continuity of care after discharge from acute COVID-19 pathway. In the future, designing studies to document long COVID-19 complications using a longitudinal design and interventions to improve follow-up care after discharge from COVID-19 acute care pathway will be necessary. In addition, there is need to evaluate using implementation science methodology the successes and failures of post-COVID condition tracking in Nigeria.

## Data Availability

The data collected in this study is available on request.

## Abbreviations

CHAI: Clinton Health Access Initiative
COVID-19: Coronavirus Disease 2019
NGT: Nominal Group Technique
NToT: National Training of Trainers
SARS-CoV-2: Severe Acute Respiratory Syndrome
SSA: Sub-Saharan Africa
WHO: World Health Organization

## References

[1] World Health Organization. Coronavirus (COVID-19) dashboard. https://covid19.who.int/. Accessed on April, 16, 2023.

[2] World Health Organization. A clinical case definition of post COVID-19 condition by Delphi consensus, 6 October 2021. https://www.who.int/publications/i/item/WHO-2019-nCoV-Post_COVID-19_condition-Clinical_case_definition-2021.1. Accessed on April, 16, 2023.

[3] World Health Organization. Coronavirus disease (COVID-19): Post-COVID-19 condition. https://www.who.int/news-room/questions-and-answers/item/coronavirus-disease-(covid-19)-post-covid-19-condition. Accessed on April, 16, 2023.

[4] Wulf Hanson S, Abbafati C, Aerts JG, Al-Aly Z, Ashbaugh C, Ballouz T (2022). Estimated global proportions of individuals with persistent fatigue, cognitive, and respiratory symptom clusters following symptomatic COVID-19 in 2020 and 2021. JAMA.

[5] Rochmawati E, Iskandar AC, Kamilah F (2022). Persistent symptoms among post-COVID-19 survivors: a systematic review and meta-analysis. J Clin Nurs.

[6] Maglietta G, Diodati F, Puntoni M, Lazzarelli S, Marcomini B, Patrizi L, Caminiti C (2022). Prognostic factors for Post-COVID-19 syndrome: a systematic review and Meta-analysis. J Clin Med.

[7] Dryden M, Mudara C, Vilka C, Blumberg L, Mayet N, Cohen C (2022). Post-COVID-19 condition 3 months after hospitalization with SARS-CoV-2 in South Africa: a prospective study. Lancet Global Health.

[8] Kreyenbuhl J, Nossel IR, Dixon LB (2009). Disengagement from mental health treatment among individuals with schizophrenia and strategies for facilitating connections to care: a review of the literature. Schizophr Bull.

[9] Kendall CE, Fitzgerald M, Donelle J, Kwong JC, Galanakis C, Boyd R (2020). A cross-sectional study of prolonged disengagement from clinic among people with HCV receiving care in a low-threshold, multidisciplinary clinic. Can Liver J.

[10] Ogueji IA, Ogunsola OO, Abdalla NM, Helmy M. Mistrust of the Nigerian health system and its practical implications: qualitative insights from professionals and non-professionals in the Nigerian health system. J Public Health. 2023;1–12. 10.1007/s10389-022-01814-z.

[11] Aregbeshola BS (2016). Out-of-pocket payments in Nigeria. Lancet.

[12] Ashipala DO, Tomas N, Costa Tenete G. Barriers and facilitators affecting the uptake of COVID-19 vaccines: a qualitative perspective of frontline nurses in Namibia. SAGE Open Nurs. 2023;9. 10.1177/23779608231158419. 10.1177/23779608231158419 PMC9969425 36861054

[13] Lieneck C, Herzog B, Krips R (2021). Analysis of facilitators and barriers to the delivery of Routine Care during the COVID-19 global pandemic: a systematic review. Healthc (Basel).

[14] Obach A, Cabieses B, Vezzani F, Robledo C, Blukacz A, Vial P (2023). Perceived barriers and facilitators for adhering to COVID-19 preventive measures in Chile: a qualitative study in three large cities. BMC Infect Dis.

[15] Allen J, Dyas J, Jones M. (2004) Building consensus in health care: a guide to using the nominal group technique. British Journal of Community Nursing, 2004; 9 (3): 110–114. 10.12968/bjcn.2004.9.3.12432 15028996

[16] Abbas S, Kermode M, Khan MD, Denholm J, Adetunji H, Kane S (2023). What makes people with chronic illnesses discontinue treatment? A practice theory informed analysis of adherence to treatment among patients with drug-resistant tuberculosis in Pakistan. Int J Health Policy Manag.

[17] Rachlis B, Naanyu V, Wachira J (2016). Identifying common barriers and facilitators to linkage and retention in chronic disease care in western Kenya. BMC Public Health.

[18] Castro-Avila AC, Jefferson L, Dale V, Bloor K (2021). Support and follow-up needs of patients discharged from intensive care after severe COVID-19: a mixed-methods study of the views of UK general practitioners and intensive care staff during the pandemic’s first wave. BMJ Open.

[19] Wallace AS, Raaum SE, Johnson EP, Presson AP, Allen CM, Elliott M, et al. Impact of COVID-19 visitation policies and hospital capacity on discharge readiness in medicine patients. Discov Health Syst. 2023;2(45). 10.1007/s44250-023-00060-8. 10.1007/s44250-023-00060-8 PMC10689550 38045443

[20] Li R, Geng J, Liu J, Wang G, Hesketh T (2022). Effectiveness of integrating primary healthcare in aftercare for older patients after discharge from tertiary hospitals-a systematic review and meta-analysis. Age Ageing.

[21] Starfield B, Shi L, Macinko J (2005). Contribution of primary care to health systems and health. Milbank Q.

[22] Elugbadebo O, Ojagbemi A, Adefolarin A, Gureje O. Access and Discontinuity of Care at an Outpatient Mental Health Service for older people in South Western Nigeria. Community Ment Health J. 2021;81518–24. 10.1007/s10597-020-00768-4. Epub 2021 Jan 7. PMID: 33411083. 10.1007/s10597-020-00768-4 33411083

